# Transcriptomic Evaluation of Hollow Microneedles-Mediated Drug Delivery for Rheumatoid Arthritis Therapy

**DOI:** 10.3390/bios15120782

**Published:** 2025-11-27

**Authors:** Zhibo Liu, Xiaotong Li, Suhang Liu, Yijing Cai, Xingyuan Xu, Siqi Gao, Chuanjie Yao, Linge Wang, Xi Xie, Yanbin Cai, Lelun Jiang, Jing Liu, Mingqiang Li, Yan Li, Xinshuo Huang, Huijiuan Chen

**Affiliations:** 1 State Key Laboratory of Optoelectronic Materials and Technologies, Guangdong Province Key Laboratory of Display Material and Technology, School of Electronics and Information Technology, Sun Yat-sen University, Guangzhou 510006, China; 2 Department of Cardiology, The First Affiliated Hospital of Jinan University, Guangzhou 510630, China; 3 Guangdong Provincial Key Laboratory of Functional and Intelligent Hybrid Materials and Devices, South China Advanced Institute for Soft Matter Science and Technology School of Emergent Soft Matter, State Key Laboratory of Luminescent Materials and Devices, Guangdong Basic Research Center of Excellence for Energy and Information Polymer Materials, South China University of Technology, Guangzhou 510640, China; 4 Zhujiang Hospital of Southern Medical University, Southern Medical University, Guangzhou 510280, China; 5 Guangdong Provincial Key Laboratory of Sensor Technology and Biomedical Instrument, School of Biomedical Engineering, Sun Yat-sen University, Shenzhen 518107, China; 6 Institute of Precision Medicine, The First Affiliated Hospital, Sun Yat-sen University, Guangzhou 510080, China; 7 Laboratory of Biomaterials and Translational Medicine, Center for Nanomedicine, The Third Affiliated Hospital, Sun Yat-sen University, Guangzhou 510630, China

**Keywords:** hollow microneedle, drug delivery, rheumatoid arthritis, transcriptomic, targeted therapy

## Abstract

Microneedle array-based drug delivery offers a minimally invasive and safe approach for breaching the skin barrier, enabling localized and targeted treatment—an advantage particularly valuable in chronic condition management, such as rheumatoid arthritis (RA). RA presents a multifaceted pathophysiology, often necessitating long-term pharmacological management. However, conventional oral administration may lead to systemic drug distribution, increasing the likelihood of adverse effects, and ultimately undermining therapeutic efficacy. In this study, a hollow microneedle array was employed for effective delivery of Tofacitinib and the antioxidant N-acetylcysteine (NAC). A comprehensive evaluation was conducted across multiple levels, in which inflammation and cartilage degradation were assessed histologically using hematoxylin-eosin (H&E) and Safranin O–Fast Green staining. Radiologically, micro-computed tomography (micro-CT) was employed to visualize bone structure alterations. On the molecular level, enzyme-linked immunosorbent assay (ELISA) was used to quantify inflammatory cytokines and oxidative stress markers. Furthermore, differentially expressed genes and enriched signaling pathways were identified through transcriptomic profiling pre- and post-treatment. And the potential regulatory targets and mechanistic insights into the therapeutic response were elucidated through correlation analyses between gene expression profiles and pathological indicators. This study provides a mechanistic and computational basis for precision targeted therapy, validates the efficacy and safety of microneedle delivery in a rheumatoid arthritis (RA) model, and demonstrates its potential application in local drug delivery strategies.

## 1. Introduction

Rheumatoid arthritis (RA) is a systemic autoimmune disease characterized by chronic and progressive joint inflammation, with a global prevalence of approximately 0.5% to 1% [1]. The pathological hallmarks of RA include abnormal synovial hyperplasia, infiltration of inflammatory cells, pannus formation, cartilage erosion, and bone destruction, ultimately leading to irreversible joint deformity and functional impairment. These structural and functional changes impose a substantial and long-lasting burden on both individual patients and healthcare systems [2]. In recent years, therapeutic strategies have increasingly shifted toward early diagnosis, mechanism-driven intervention, and personalized treatment paradigms [3]. For chronic inflammatory conditions such as RA, treatment goals have evolved beyond symptom control to include prevention of irreversible joint damage and sustained improvement of patient quality of life.

**Figure 1:**
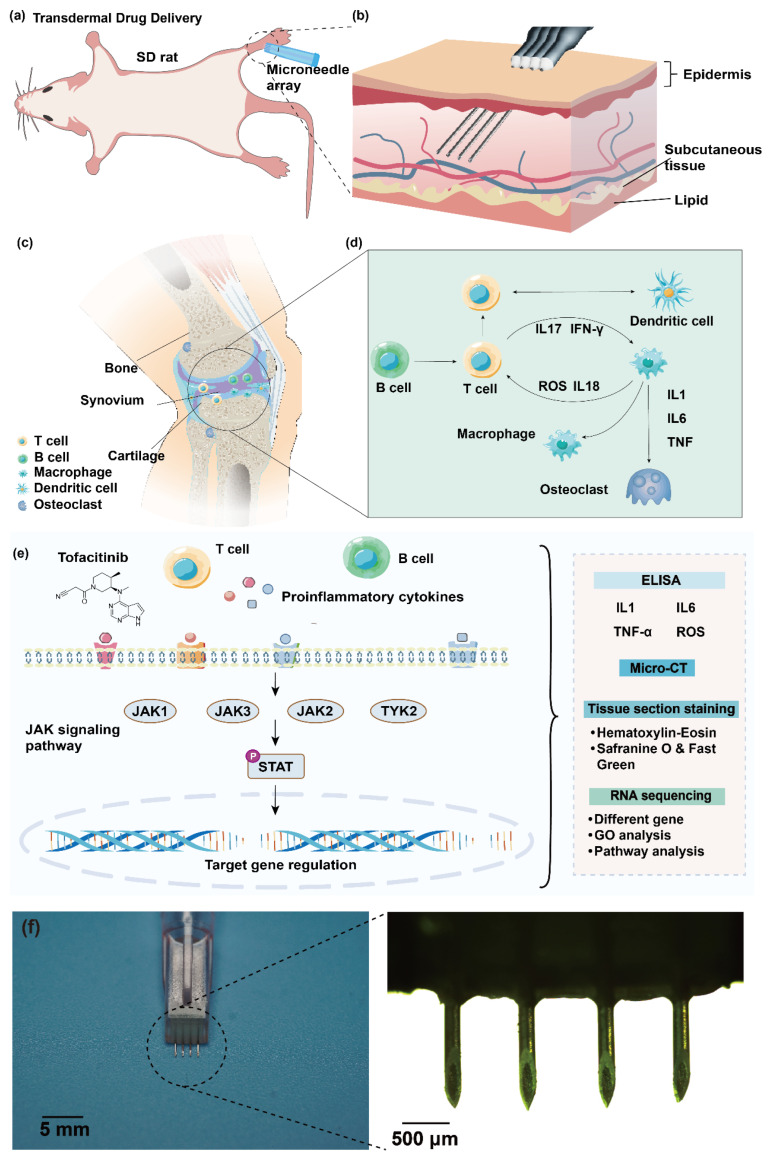
Schematic of transdermal drug delivery using hollow microneedles in SD rats and integrated evaluation strategy for RA treatment. (**a**,**b**) Hollow microneedles were used for localized transdermal delivery into the ankle joints of SD rats with collagen-induced arthritis, enabling minimally invasive penetration from the epidermis to the dermis. (**c**) RA pathology involves complex immune cell infiltration (T cells, B cells, macrophages, dendritic cells, osteoclasts) and inflammatory mediator release (e.g., IL-1, IL-6, TNF-α, IFN-γ, IL-17, ROS), resulting in synovial inflammation, cartilage erosion, and bone remodeling. (**d**) Tofacitinib targets the JAK-STAT pathway, inhibiting JAK1 and JAK3 to reduce proinflammatory cytokine signaling and immune cell activation. (**e**) Delivery of Tofacitinib and antioxidant NAC via hollow microneedles was designed to simultaneously suppress inflammation and oxidative stress. Multimodal assessments—including histology (H&E, Safranin O/Fast Green), micro-CT imaging, ELISA quantification of inflammatory markers, and RNA sequencing—were performed to evaluate therapeutic outcomes across structural, biochemical, and transcriptomic levels. (**f**) Microneedle drug administration was conducted daily from Day 8 to Day 28 post-induction.

Tofacitinib is an orally administered Janus kinase (JAK) inhibitor that targets the JAK-signal transducer and activator of transcription (JAK-STAT) signaling pathway, thereby suppressing multiple pro-inflammatory cytokines, including interleukin (IL)-6, IL-2, IL-7, IL-15, and interferons (Figure 1a,b) [2,4,5]. By modulating immune cell activation and cytokine release, it effectively inhibits synovial inflammation, T- and B-cell activity, and bone destruction [6]. Tofacitinib has been clinically validated as an effective therapy for patients who show inadequate responses to conventional synthetic disease-modifying antirheumatic drugs (csDMARDs) such as methotrexate [7], and could be used either as monotherapy or in combination [4]. Sustained administration has demonstrated efficacy in achieving disease remission and delaying structural progression, with evidence including reduced bone erosion, joint space narrowing [8,9], MRI-confirmed synovial thickening and bone marrow edema [10]. Across clinical trials, Tofacitinib shows favorable tolerability with adverse events rates comparable to biologics [11], though infection—particularly in immunocompromised patients—remains a key safety concern, warranting long-term monitoring [11]. Median treatment duration of approximately five years was suggested by drug retention studies, with discontinuation more often due to adverse events than to loss of efficacy [12].

Joint inflammation in RA is frequently accompanied by elevated oxidative stress and the excessive accumulation of reactive oxygen species (ROS), which in turn activate immune cells and perpetuate the autoimmune response. This positive-feedback loop could exacerbate inflammation, induce cellular damage, and accelerate joint destruction [13,14]. N-acetylcysteine (NAC), a well-known antioxidant, functions by promoting the synthesis of glutathione and other endogenous antioxidant molecules. In this way, the intracellular ROS accumulation could be reduced by NAC to enhance cellular antioxidant defenses and suppress lipid peroxidation induced by free radicals and peroxides—ultimately contributing to the attenuation of RA symptoms [15]. NAC administration has been demonstrated to significantly lower serum malondialdehyde levels, a biomarker of lipid peroxidation, suggesting a reduction in oxidative stress-related cellular injury in both clinical and preclinical studies. The antioxidant properties of NAC thus hold therapeutic potential in mitigating ROS-mediated joint pathology in RA [16].

In clinical practice, NAC is utilized widely owing to its favorable safety and tolerability profile, especially in disorders associated with oxidative stress [17]. Nonetheless, NAC administration should be individualized and conducted under medical supervision, with appropriate monitoring to ensure both therapeutic efficacy and safety. Due to features including ease of administration, enhanced patient compliance, and potential sustained release, transdermal drug delivery via microneedles was particularly advantageous for the long-term treatment of chronic inflammatory diseases such as RA [18,19,20]. By penetrating the stratum corneum and delivering therapeutic agents into the superficial dermis, microneedle arrays enable minimally invasive drug administration, which could enhance delivery precision while minimizing pain and tissue trauma [21]. This targeted approach allows for localized drug release at the inflamed tissue, while avoiding direct stimulation of capillaries and nociceptive nerve endings, ultimately improving patient comfort and tolerability.

Furthermore, microneedle-assisted subcutaneous delivery facilitates prolonged local drug retention and enables sustained therapeutic action at the disease site. The dense capillary network within the subcutaneous tissue supports rapid systemic absorption, thereby achieving both localized disease modulation and systemic therapeutic effects. Traditional drug delivery methods (such as oral administration) require absorption through the gastrointestinal tract, where drugs may undergo metabolic breakdown in the liver before entering systemic circulation. In contrast, microneedle-based delivery directly introduces drugs into the subcutaneous tissue, representing an emerging technology capable of achieving near-localized targeted delivery. This approach is particularly well-suited for treating conditions like arthritis that require targeted drug administration. Therefore, compared to oral administration, microneedle-based delivery bypasses first-pass hepatic metabolism, improves drug bioavailability, and reduces systemic side effects—offering a promising and patient-friendly strategy for long-term management of localized inflammatory disorders such as RA [22,23]. The development of an advanced drug delivery system that integrate targeted release, sustained pharmacological action, and local microenvironmental regulation is the key to further enhance the accuracy and safety of RA therapy [24]. Leveraging the excellent skin-penetrating capability, delivery precision, and high compliance of microneedles, a hollow microneedle array was employed for effective delivery of Tofacitinib and NAC separately—two pharmacologically complementary agents—via localized subcutaneous administration in an RA animal model. The therapeutic efficacy of this microneedle-mediated combination therapy was comprehensively assessed through histological, imaging, electrochemical, and bioinformatics analyses. Furthermore, transcriptomic profiling and machine learning algorithms were applied to uncover the underlying molecular mechanisms and identify key regulatory targets associated with treatment response. Collectively, this work aims to demonstrate the feasibility and multidimensional advantages of microneedle-based localized combination therapy for RA, providing a mechanistic and technological foundation for the developing individualized, mechanism-driven treatment strategies for chronic inflammatory diseases.

## 2. Method

### 2.1. Materials and Reagents

Tofacitinib (MW: 312.37, 1 g/vial) was purchased from Kele Biotechnology Ltd (Guangzhou, China). and dissolved in DMSO (Aladdin Biochemical Technology Co., Ltd., Shanghai, China) to prepare a 0.5653 mg/mL solution. N-acetylcysteine (NAC, 5 g/vial, MCE) was obtained from Zuoke Biotech (Guangzhou, China) and dissolved in NaOH solution to a concentration of 67.83 mg/mL. Complete Freund’s Adjuvant (CFA, 10 mL/vial) was supplied by Bixing Technology Ltd (Guangzhou, China). RNA preservation reagent (RNAwait, 100 mL) was purchased from Solarbio (Beijing, China). Other reagents included NaOH (500 g, Macklin), 95% paraformaldehyde (Macklin), PBS buffer, and 70% ethanol. Sterile PES syringe filters (0.45 μm, 30 mm) were obtained from Guangzhou Jet (Guangzhou, China). Four-pin microneedle arrays were sourced from Kangpuwo (Chengdu, China). ELISA kits for rat TNF-α, IL-1β, IL-6, and ROS were purchased from Jiangsu Meimian Biotechnology (Jiangsu, China). Deionized water used for solution preparation was obtained from Macklin. A SPECTRA MAX 190 microplate reader (Molecular Devices, Shanghai, China) was used for ELISA measurements. Histological sections were observed using an Olympus BX43 microscope (Olympus, Tokyo, Japan). All reagents were used as received unless otherwise specified.

### 2.2. Establishment of RA Model in Rats

Male Sprague-Dawley rats (150–180 g, 6–8 weeks old) were obtained from the Experimental Animal Center of Sun Yat-sen University (license number provided). Animals were housed under specific pathogen-free (SPF) conditions (22 ± 1 °C, 50 ± 5% humidity) with ad libitum access to sterilized chow and purified water for 7 days prior to experiments.

To induce RA, rats were anesthetized with 4% isoflurane (*v*/*v*) in oxygen (1.5 L/min). The plantar surface of the right hind paw was alternately disinfected with 0.5% povidone-iodine and 75% ethanol. Using a sterile 1 mL syringe fitted with a 27 G needle, 0.1 mL of Complete Freund’s Adjuvant (CFA, containing 10 mg/mL heat-inactivated Mycobacterium tuberculosis, Sigma-Aldrich, F5881) was injected intradermally. Injection sites were gently compressed for 30 s post-injection to prevent reflux. Animals were monitored and returned to their cages after confirming the absence of local hematoma.

### 2.3. Preparation of Tofacitinib Solution

Precisely 5.66 mg of Tofacitinib was weighed into a 15 mL conical tube (Corning, New York, NY, USA), followed by the addition of 10 mL of phosphate-buffered saline (PBS) containing 20% (*w*/*v*) hydroxypropyl-β-cyclodextrin (HP-β-CD). The mixture was vortexed for 30 s using Vortex-Genie (Scientific Industries, Inc., New York, NY, USA) in 22,500 rpm. To promote complete dissolution, the solution was incubated at 37 °C for 10 min and subjected to three cycles (40 kHz, 200 W, 3 × 5 min) of ultrasonication (Branson 3800, Emerson Electric Co., Danbury, NC, USA). The final solution was sterile-filtered through a 0.22 μm PVDF membrane (Guangzhou Jet, Guangzhou, China), aliquoted into sterile 2 mL cryovials (NEST) to prepare a 0.5653 mg/mL Tofacitinib solution. This solution was administered subcutaneously at 0.1 mL/100 g body weight, achieving precise dosing of 0.05653 mg/100 g.

### 2.4. Preparation of Acetylcysteine Solution

Based on clinical pharmacokinetic data and body surface area, the subcutaneous drug delivery dose of NAC for rats is 6.783 mg/100 g. Briefly, 678.3 mg of high-purity NAC powder (≥99.0%, HPLC-grade) was accurately weighed (Sartorius CPA225D, Göttingen, German, ±0.01 mg) and dissolved in 10 mL of pre-warmed (37 °C) 0.9% saline. The final solution was sterile-filtered (0.22 μm PVDF membrane, Millipore, Burlington, MA, USA), aliquoted into 1 mL light-proof EP tubes (Axygen Biosciences, Inc., New York, NY, USA), wrapped in double-layer aluminum foil (0.02 mm), and stored at 4 °C. Prior to use, aliquots were equilibrated to room temperature (25 °C), vortexed for 5 min (2000 rpm) and sterility (TSA incubation at 37 °C for 48 h). The injection volume was 0.1 mL/100 g body weight.

### 2.5. Animal Experimental Design

In a rat model of RA, effective delivery of tofacitinib and NAC was achieved using hollow microneedles to validate the therapeutic potential of the local drug delivery system. Male SD rats were randomly assigned to five groups: healthy control, RA model (day 18), RA model (day 28), RA + Tofacitinib, and RA + NAC. Drug interventions were initiated on day 8 post-RA induction, corresponding to the onset of visible inflammatory symptoms. On day 18, animals in the RA-18d group were sacrificed, and ankle joint tissues were harvested for histological staining, micro-CT analysis, transcriptome profiling, and serum ELISA. On day 28, the same procedures were performed for all remaining groups. Body weight and ankle thickness were recorded weekly throughout the study.

### 2.6. Histological Analysis

Right ankle joints, including synovial cavities and surrounding periarticular tissues, were excised and fixed in 4% neutral-buffered formalin (pH 7.4) at 4 °C for 24 h. Samples were then decalcified in 10–17% EDTA solution (pH 7.2) until adequately softened. Following graded ethanol dehydration and xylene clearing, tissues were sagittally embedded in paraffin. Serial paraffin sections (4 μm thick) were prepared and subjected to hematoxylin and eosin (H&E) staining for general histopathological evaluation and Safranin O/Fast Green staining to assess cartilage integrity.

### 2.7. Micro-CT Sample Preparation and Analysis

Following euthanasia, right ankle joints were aseptically dissected and fixed in 4% paraformaldehyde (in 0.1 M PBS, pH 7.4) at 4 °C. After fixation, samples were rinsed three times with PBS (15 min each) and subsequently transferred to 70% ethanol for preservation at 4 °C until scanning. High-resolution micro-computed tomography (micro-CT) was performed, and three-dimensional reconstruction of joint structures was conducted using CTvol V6.0 software. Quantitative analysis included: (1) bone volume fraction (BV/TV); (2) trabecular thickness (Tb.Th) and trabecular separation (Tb.Sp); (3) subchondral bone plate porosity; and (4) joint space width (JSW).

### 2.8. Serum Collection for ELISA

Whole blood was obtained via abdominal aorta puncture and allowed to clot at room temperature (25 °C) in the dark for 30 min. Samples were then centrifuged at 3000× *g* for 15 min at 4 °C (Eppendorf, Shanghai, China). The resulting serum was carefully aliquoted into sterile cryovials (Corning, New York, NY, USA) and stored at −80 °C until ELISA analysis.

### 2.9. Transcriptomic Sample Preparation

Ankle joints were aseptically harvested and carefully dissected to remove surrounding skin and muscle tissues. Samples were immediately immersed in RNA stabilization solution (RNAwait, Beyotime, R0020) to preserve RNA integrity and subsequently stored at −80 °C until transcriptome sequencing.

### 2.10. Histological Data Quantification

Histological images were analyzed using ImageJ software (Version: 2.15.0). Brightness and contrast were adjusted, and the Threshold and Analyze tools were applied to quantify positive staining areas and perform cell counting. Cartilage thickness was measured using Image-Pro Plus (Version: 2.15.0) by manually delineating the cartilage boundaries and calculating the average thickness across multiple regions per sample.

### 2.11. Pathological–Genomic Association Analysis

Eight key pathological indicators were quantified: (1) Safranin O/Fast Green staining ratio (100×/200×); (2) cartilage thickness; (3) subchondral cell density; and (4) serum concentrations of IL-1β, IL-6, TNF-α, and ROS. A random forest classifier (scikit-learn v1.2.2; n_estimators = 100, random_state = 42) was employed to evaluate the relative importance of 23 candidate genes (e.g., *TNFRSF14*, *FCGR2B*, *PTPRC*). Categorical group labels were encoded, and gene expression data were standardized using Z-score normalization. Feature importance was calculated based on Gini impurity and visualized as horizontal bar plots. To construct a multi-omic association network, a random forest regression model (n_estimators = 200, max_depth = 10) was applied. Genes significantly associated with inflammatory mediators (importance > 0.15, FDR < 0.05) were identified using an independent test set (test_size = 0.2).

### 2.12. Statistical Analysis

The data were calculated and expressed as the mean ± standard error (SE). The differences between the two compared groups were determined by one-way ANOVA analysis. *p*-values < 0.05 indicated that the results were considered statistically significant. * *p* < 0.05, ** *p* < 0.01, and *** *p* < 0.001.

## 3. Result

For drug delivery, a four-needle commercially available microneedles (34 G × 1.2 mm) were employed (Figure 1f) The use of these microneedles streamlined the drug delivery process by reducing the need for extensive sterile processing, and enhancing biosafety. The RA model was established by inducing adjuvant-induced arthritis in Sprague-Dawley rats through subcutaneous injection of complete Freund’ s adjuvant (CFA) into the right hind paw. During therapeutic administration, the microneedle array was first assembled with a 1 mL medical screw-cap syringe. Subsequently, the required drug dosage, calculated based on the rat’ s body weight, was precisely aspirated into the syringe. The microneedle array was then inserted at a perpendicular angle into the subcutaneous tissue on the medial aspect of the ankle joint. Upon penetration of the skin’s stratum corneum and epidermis, the drug was delivered to the targeted subcutaneous area by advancing the syringe plunger. This method ensures effective penetration of the skin’s barrier layers, enabling efficient and precise drug delivery to the intended site.

For systematical evaluation of the therapeutic efficacy of JAK inhibition and ROS scavenging via microneedle-assisted drug delivery, animals were randomly assigned to five experimental groups: (1) a healthy control group, (2) an RA 18-day group, (3) an RA 28-day group, (4) a Tofacitinib treatment group, and (5) an NAC treatment group. The healthy control group was used as the baseline for physiological and histopathological parameters to minimize inter-animal variability. The RA 18-day and RA 28-day groups, which received CFA without therapeutic intervention, were euthanized at days 18 and 28 post-induction, respectively, to characterize disease progression at early and advanced stages. The Tofacitinib and NAC treatment groups received daily subcutaneous administration of their respective drugs using the microneedle system from day 8 to day 28 post-induction thereby enabling comparative assessment of the effects of JAK-STAT pathway inhibition and antioxidant therapy on RA pathophysiology.

The selection of timepoints was based on the dynamic progression of inflammation commonly observed in RA models. Initial joint swelling typically appears around day 3 following adjuvant induction, with a secondary inflammatory surge generally occurring after day 8. From days 10 to 18, a delayed-type hypersensitivity response of animals was observed, with contralateral limb swelling and the appearance of nodules on the ears and tail. This phase transitions into polyarthritis between days 18 and 25, after which the pathological progression tends to stabilize around day 28.

At each predefined timepoint, animals in the respective groups underwent comprehensive assessments encompassing both morphological and molecular endpoints. Specifically, the conducted evaluations included: (1) Histological analysis using hematoxylin and eosin (HE) staining and safranin O/fast green staining to evaluate synovial inflammation, pannus formation, and cartilage degradation; (2) Micro-computed tomography (micro-CT) to visualize structural alterations in subchondral bone and joint architecture; (3) Enzyme-linked immunosorbent assay (ELISA) to quantify circulating levels of key pro-inflammatory cytokines (IL-1β, IL-6, TNF-α) and ROS, serving as indicators of systemic inflammation and oxidative stress; (4) Transcriptomic sequencing of synovial tissue samples to identify differentially expressed genes associated with JAK-STAT signaling and oxidative stress pathways, thereby uncovering potential molecular mechanisms underlying the observed therapeutic responses.

### 3.1. Histopathological Observation and Cytokine Analysis

Histological evaluation represents a critical component in the pathological assessment of RA, offering direct insights into how therapeutic interventions modulate disease mechanisms at the tissue level. In this study, hematoxylin and eosin (HE) staining and safranin O/fast green staining were employed to quantitatively assess synovial inflammation and cartilage matrix degradation, respectively. HE staining was utilized to evaluate morphological alterations in the synovial membrane, including architectural disruption and the extent of inflammatory cell infiltration. In parallel, safranin O/fast green staining was used to assess cartilage degeneration by distinguishing proteoglycans (stained red) from collagen fibers (stained green), allowing for precise quantification of proteoglycan loss based on the percentage of positively stained area.

**Figure 2:**
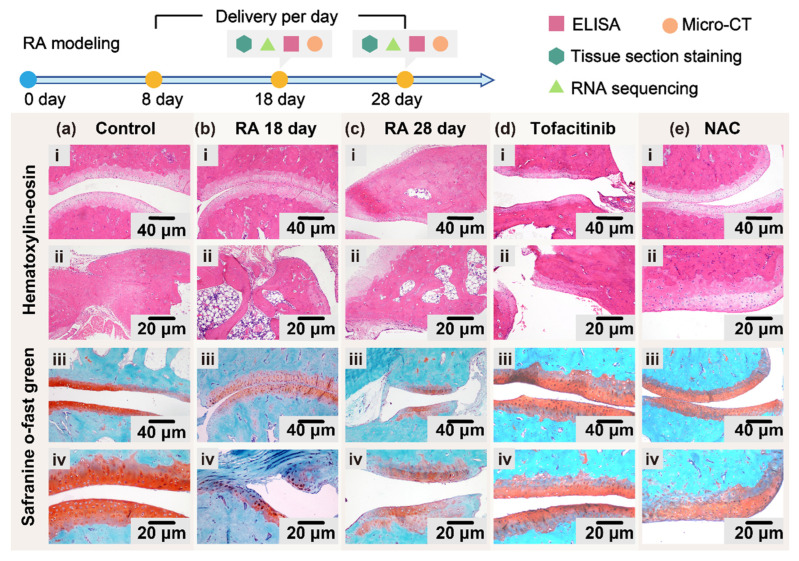
Histological evaluation of ankle joint pathology in RA rats and therapeutic response to hollow microneedle-based treatment. Schematic timeline of RA induction, drug administration via hollow microneedles, and sample collection for histology, ELISA, micro-CT, and transcriptomic analysis. (**a**–**e**, i,ii) Hematoxylin–eosin (H&E) staining and (iii,iv) Safranin O–Fast Green staining of ankle joints across experimental groups. (**a**) Control group exhibits intact cartilage architecture, smooth joint surface, and normal synovial structure. (**b**) RA Day 18 group shows significant joint space narrowing or obliteration, accompanied by synovial hyperplasia and localized fibrosis. (**c**) RA Day 28 group displays further exacerbation of these changes, including pronounced inflammatory cell infiltration and structural disorganization. (**d**) Tofacitinib treatment partially restores synovial morphology, though mild hyperplasia with villous projections remains visible. (**e**) NAC treatment preserves joint space and cartilage integrity to a greater extent, with reduced synovial fibrosis compared to the RA group.

To investigate pathological changes across experimental groups, histological analyses were performed on the right hind ankle joints and surrounding soft tissues. The specimens were harvested and fixed in 4% neutral-buffered formalin at 4 °C for 24 h. Following fixation, samples underwent decalcification until the osseous matrix softened adequately, then were subjected to graded ethanol dehydration and xylene clearing. Paraffin embedding was performed in the sagittal orientation, and serial sections of 4 μm thickness were prepared using a microtome. The Section 3 were stained with HE and safranin O/fast green for morphological assessment and matrix integrity evaluation (Figure 2a–e).

In the healthy control group (Figure 2a), histological sections at both 100× and 200× magnifications revealed a smooth, intact articular cartilage surface with a well-defined cartilage layer and regularly arranged chondrocytes. No signs of inflammatory cell infiltration or degenerative changes were observed. The cartilage matrix appeared homogeneous and continuous, without evidence of cracking or fragmentation, and the underlying subchondral bone maintained a normal, organized architecture—indicating a stable joint environment free from pathological disruption.

In the RA model groups, joint pathology progressively worsened over time. By day 18 post-induction (Figure 2b), narrowing or even obliteration of the joint space was observed, accompanied by localized joint fibrosis. Chondrocyte disorganization was apparent in certain regions, along with mild inflammatory infiltration within the cartilage layer. Early matrix degradation and distorted chondrocyte morphology were also evident, marking the onset of inflammation-induced cartilage injury. By day 28 (Figure 2c), persistent joint fibrosis and pronounced tissue alterations suggested progression toward chronic joint destruction. These findings reflected an ongoing inflammatory process with sustained cartilage damage and limited spontaneous recovery. In the Tofacitinib treatment group, histological sections at 100× magnification (Figure 2d) exhibited characteristic synovial hyperplasia, including finger-like projections extending into the joint cavity. Nevertheless, the cartilage structure showed notable improvement compared with the untreated RA model groups. The articular surface remained relatively smooth, matrix degradation was mild, and inflammatory infiltration was visibly reduced. The cartilage layer exhibited partial restoration of structure, with more orderly chondrocyte arrangement, suggesting that JAK inhibition contributed to attenuation of inflammation and preservation of cartilage integrity during disease progression. In the NAC treatment group, a narrowed but distinguishable joint space was observed, and the cartilage structure remained largely intact (Figure 2e). Chondrocytes were arranged in a regular pattern, and only mild matrix degradation was noted. Inflammatory infiltration was substantially reduced, and the overall histological architecture was well preserved. These findings highlight the cartilage-protective potential of NAC, likely mediated through its antioxidative and anti-inflammatory properties, and suggest a capacity to promote tissue repair within the inflamed joint microenvironment.

Safranin O–Fast Green staining further revealed pathological changes in the cartilage extracellular matrix and subchondral bone across groups. In the healthy control group, strong and uniform safranin O staining indicated a high content of proteoglycans within the cartilage matrix, while fast green staining outlined an intact and well-organized subchondral bone structure—suggesting normal cartilage metabolism and bone integrity (Figure 2a). In contrast, by day 18 post-RA induction (Figure 2b), a marked reduction in safranin O staining intensity was observed relative to the healthy controls, reflecting early loss of proteoglycans and the initiation of cartilage matrix degradation. Concurrently, an increased area of fast green staining suggested elevated subchondral bone density, indicative of early-stage bone remodeling triggered by joint inflammation. By day 28, these pathological features became more pronounced. Safranin O staining was further diminished, consistent with progressive proteoglycan depletion and ongoing matrix degeneration. These findings collectively illustrate the gradual deterioration of joint cartilage and subchondral bone architecture over time in the untreated RA model (Figure 2c).

Pharmacological interventions with Tofacitinib and N-acetylcysteine (NAC) showed differential protective effects on joint structure. In the Tofacitinib-treated group (Figure 2d), partial recovery of safranin O staining was observed compared with the RA day 28 group, suggesting improved retention or restoration of proteoglycan content. Meanwhile, fast green staining patterns indicated relatively stable subchondral bone architecture, pointing to a moderate bone-preserving effect. In the NAC-treated group (Figure 2e), safranin O staining intensity closely resembled that of the healthy control group, reflecting better preservation of cartilage matrix integrity and proteoglycan content. Additionally, fast green staining revealed uniform subchondral bone structure with minimal evidence of remodeling or structural disruption, and inflammatory infiltration appeared reduced. These observations suggest that NAC may exert both antioxidative and anti-inflammatory effects contributing to enhanced joint protection and cartilage repair. In summary, the Safranin O–Fast Green staining results confirmed that RA induction successfully recapitulated typical histopathological features of cartilage degradation and bone remodeling, with progressive matrix loss evident by day 28. Both Tofacitinib and NAC mitigated cartilage destruction and preserved structural integrity to varying degrees, with NAC exhibiting a relatively stronger chondroprotective effect under the current experimental conditions.

Additionally, paw thickness was quantitatively assessed as an objective marker of joint inflammation. Increases in paw thickness are indicative of hallmark pathological features of RA, including synovitis, joint effusion, and periarticular soft tissue swelling. Serial measurements of paw thickness in each experimental group provided a means to dynamically monitor disease progression and evaluate the anti-inflammatory efficacy of therapeutic interventions. This parameter offered excellent temporal resolution, ease of operation, and non-invasive applicability. The results demonstrated that the RA model group exhibited significantly increased paw swelling compared to the healthy control group, consistent with active joint inflammation. These observations were further corroborated by histological staining: quantification of safranin O–fast green-positive regions revealed the smallest cartilage-stained area in the untreated RA model group, reflecting severe proteoglycan loss and matrix degradation. In contrast, both Tofacitinib- and NAC-treated groups displayed notably larger safranin O–positive areas, indicating preservation of cartilage matrix components and chondroprotective effects. Regarding inflammatory cell infiltration, histological analysis revealed that both treatment groups exhibited markedly reduced immune cell presence within the synovial membrane and adjacent joint tissues. Furthermore, quantitative measurements of cartilage thickness demonstrated that Tofacitinib and NAC interventions effectively helped maintain cartilage structural integrity and improved subchondral bone morphology compared with the untreated RA group. Collectively, these findings indicate that both Tofacitinib and NAC exert therapeutic effects in mitigating joint inflammation and structural damage in RA. Their capacity to preserve cartilage matrix, reduce inflammatory infiltration, and stabilize joint architecture supports their potential utility as disease-modifying agents in the treatment of arthritis.

**Figure 3:**
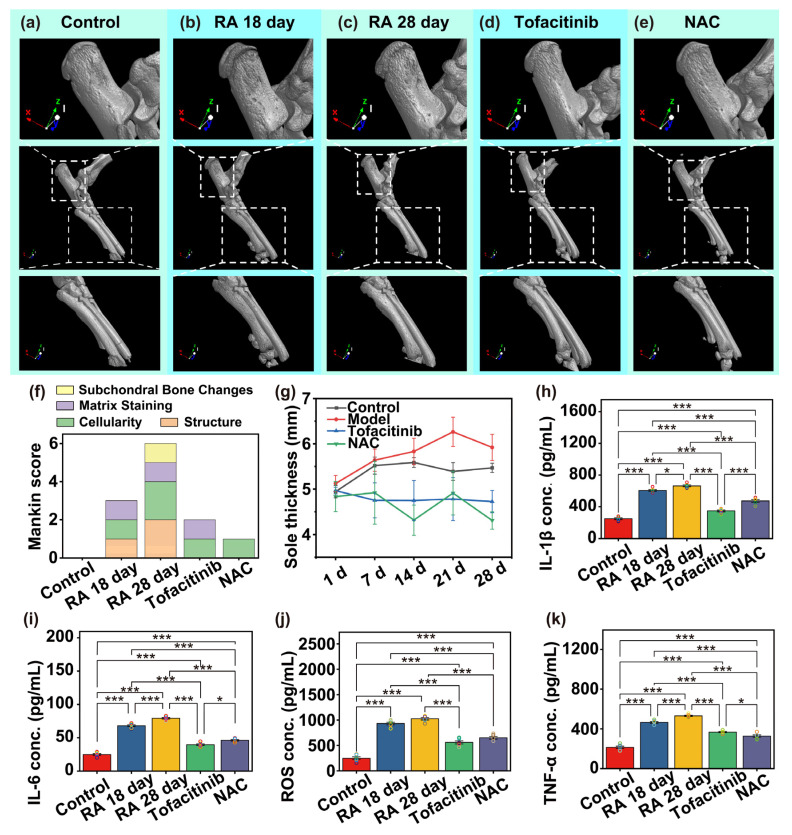
Micro-CT and biochemical assessments of joint pathology and treatment efficacy in RA rats. (**a**–**e**) Representative micro-CT images of the calcaneus and phalanges from (**a**) healthy control, (**b**) RA Day 18, (**c**) RA Day 28, (**d**) Tofacitinib-treated, and (**e**) NAC-treated groups. Progressive joint deformity and bone erosion were observed in RA groups, while both treatments alleviated structural deterioration. (**f**) Histopathological evaluation using Mankin scoring, encompassing structural integrity, cellularity, matrix staining, and subchondral bone changes, highlights progressive degeneration in RA and partial recovery with Tofacitinib or NAC. (**g**) Quantitative analysis of paw thickness over time reveals significant swelling in RA rats, which was attenuated in drug-treated groups. (**h**–**k**) ELISA results showing serum levels of inflammatory and oxidative stress markers: (**h**) IL-1β, (**i**) IL-6, (**j**) ROS, and (**k**) TNF-α. Elevated cytokine levels in RA groups were significantly reduced following treatment, indicating mitigation of systemic inflammation. N = 3 samples per group. Data were presented as mean ± SEM. *p* values were analyzed by one-way ANOVA analysis of variance. * *p* < 0.05, and *** *p* < 0.001.

Micro-CT analysis was conducted to further elucidate bone microstructural changes across different experimental groups (Figure 3a–e). As a high-resolution, three-dimensional imaging modality, micro-CT enables noninvasive and quantitative assessment of bone architecture, making it particularly suitable for evaluating arthritis-associated bone alterations. Following CFA injection, arthritis symptoms first appeared in the limb where CFA was injected, subsequently spreading to the contralateral limb and all four limb joints. In our experiment, we induced the arthritis model by administering CFA via sub-plantar injection in rats, with arthritis symptoms earliest appearing in the ankle joint region. Therefore, to precisely evaluate the localized therapeutic effects of MN-based drug delivery in the affected joint, and to ensure the accuracy of subsequent histological and micro-CT assessments, all MN administration procedures and related evaluations were concentrated in the ankle joint area. At the experimental endpoint, after euthanizing the animals, the right hindlimb ankle joint was dissected under aseptic conditions. Surplus soft tissue surrounding the joint was removed while preserving the intact joint capsule and talus-tibia-fibula complex. Later. key morphometric parameters—including bone volume fraction (BV/TV), trabecular thickness, trabecular separation, subchondral bone plate porosity, and joint space width—were analyzed to quantify bone loss and assess the therapeutic efficacy of pharmacological interventions.

At the end of the experiment, animals were euthanized under anesthesia, and the right hind ankle joints were aseptically dissected within a biosafety cabinet. Surrounding soft tissues were carefully removed to expose the articulation while preserving the integrity of the joint capsule and talocrural structures. Throughout tissue preparation, specimens were continuously moistened with physiological saline to prevent dehydration, which could interfere with image acquisition and data accuracy. Samples were subsequently fixed in 4% paraformaldehyde (PFA) prepared in 0.1 M phosphate-buffered saline (PBS, pH 7.4) at 4 °C for 24 h. Following fixation, tissues were rinsed three times in PBS (15 min per rinse), and then transferred into 70% ethanol for storage at 4 °C under constant temperature and humidity conditions until micro-CT scanning was performed. Three-dimensional reconstructions and quantitative morphometric analyses were performed using CTvol V6.0 software. The following parameters were extracted to evaluate bone integrity and joint damage: (1) Bone volume fraction (BV/TV)–representing the ratio of bone volume to total volume, indicative of overall bone mass; (2) Trabecular thickness and trabecular separation–characterizing the density and spatial distribution of trabecular bone; (3) Subchondral bone plate porosity–reflecting the structural deterioration of the load-bearing interface; (4) Joint space width–serving as an index for cartilage erosion and overall joint integrity.

Analysis of the micro-CT images and corresponding quantitative data revealed distinct alterations in bone surface morphology and microarchitecture across the experimental groups. In the RA 18-day (Figure 3b) and RA 28-day (Figure 3c) model groups, the articular surfaces of the calcaneus and phalanges appeared markedly rougher than those observed in the healthy control and treatment groups. Among all groups, the RA 18-day group exhibited the highest bone surface-to-volume ratio, indicating pronounced surface irregularity and early-stage erosive damage. The RA 28-day group also showed elevated surface roughness, though to a slightly lesser extent, suggesting that joint erosion increased with disease progression but may stabilize or decelerate in later stages. In addition, both RA model groups displayed significantly reduced bone volume fraction (BV/TV) relative to other groups, reflecting extensive trabecular bone loss associated with chronic inflammation. These findings are consistent with arthritis-induced osteolytic activity and reduced bone mass. In contrast, the Tofacitinib and N-acetylcysteine (NAC) treatment groups exhibited considerable improvement in trabecular bone structure. Both interventions significantly increased BV/TV compared with the untreated model group, with values approaching those of the healthy control group (Figure 3f,g). These results indicate that both Tofacitinib and NAC effectively mitigated bone loss and helped preserve bone volume under arthritic conditions. Collectively, these findings suggest that pharmacological intervention with Tofacitinib or NAC supports the maintenance and remodeling of bone microarchitecture, potentially through inhibition of inflammation-mediated bone resorption and promotion of structural integrity in RA.

Furthermore, analysis of trabecular thickness (Tb.Th) revealed that both the RA 18-day and RA 28-day model groups exhibited significantly thinner trabeculae compared to the Tofacitinib and N-acetylcysteine (NAC) treatment groups (Figure 3i), reflecting the impact of chronic inflammation on trabecular bone atrophy. Notably, no significant differences in trabecular separation (Tb.Sp) were observed among the groups, suggesting that the observed bone loss was primarily attributable to a reduction in trabecular thickness rather than alterations in trabecular spacing or distribution. Collectively, the micro-CT findings demonstrated substantial structural degradation of bone tissue in the RA disease model, as evidenced by surface irregularity, reduced bone volume fraction, and thinning of trabeculae. In contrast, animals receiving microneedle-mediated delivery of Tofacitinib or NAC showed partial restoration of bone microarchitecture and attenuation of bone loss. These results provide robust morphological evidence supporting the bone-protective effects of localized pharmacological intervention. Moreover, they underscore the potential of microneedle-based drug delivery to modulate bone metabolism, offering a promising strategy for the prevention and treatment of inflammatory arthritis–induced bone loss.

Subsequently, a comprehensive analysis was conducted integrating joint tissue structural scoring, physiological index measurements, and immune organ weight statistics to further assess disease severity and treatment efficacy. Among these parameters, the standardized Mankin scoring system for arthritis was employed to quantitatively evaluate cartilage degeneration. This widely recognized histopathological grading system encompasses four major criteria: (1) cartilage surface structural integrity, (2) chondrocyte arrangement and morphology, (3) matrix staining intensity (reflecting proteoglycan content), and (4) subchondral bone alterations. It serves as a core indicator for assessing RA progression and the therapeutic impact of pharmacological interventions. Mankin scoring was applied to evaluate cartilage sections from the ankle joints of rats in each experimental group, enabling objective comparison of cartilage preservation across treatments. As illustrated in Figure 3f, the RA 18-day and RA 28-day model groups exhibited significantly elevated scores, reflecting advanced cartilage degeneration and joint structural damage. In contrast, both Tofacitinib and NAC treatment groups demonstrated substantially lower Mankin scores, indicative of attenuated cartilage damage. Notably, the NAC-treated group achieved the lowest scores among all pathological groups, suggesting superior chondroprotective efficacy under the experimental conditions.

To evaluate the systemic inflammatory and oxidative stress responses associated with RA, enzyme-linked immunosorbent assays (ELISA) were performed on blood samples collected from each experimental group. Quantification focused on key pro-inflammatory cytokines, includingIL-1β, IL-6, tumor necrosis factor-α (TNF-α), and reactive oxygen species (ROS) (Figure 3h–k). These molecular biomarkers are widely recognized as critical indicators of RA disease activity and serve as valuable parameters for assessing the efficacy of therapeutic interventions. The ELISA results revealed that both Tofacitinib and NAC treatments significantly suppressed inflammatory cytokine production and oxidative stress compared to the untreated RA model group. Specifically, serum IL-1β concentrations in the treatment groups remained below 500 pg/mL, with the Tofacitinib group exhibiting the lowest IL-1β level at 364 pg/mL, approaching values observed in the healthy control group (Figure 3h). These findings suggest that Tofacitinib, by inhibiting Janus kinase-signal transducer and activator of transcription (JAK-STAT) signaling, effectively downregulates IL-1β expression. Simultaneously, NAC mitigates oxidative stress and promotes ROS scavenging, contributing to suppression of fibroblast-like synoviocyte (FLS) activation. Taken together, these data underscore a tight mechanistic link among IL-1β expression, JAK signaling activation, and ROS-mediated inflammation, and further demonstrate the dual anti-inflammatory and antioxidative effects of microneedle-delivered Tofacitinib and NAC.

Moreover, treatment with Tofacitinib and NAC significantly attenuated oxidative stress in the RA model. Quantitative ELISA data showed that reactive oxygen species (ROS) levels were reduced by approximately 40% in both treatment groups relative to the untreated RA group (Figure 3j), suggesting efficient elimination of excess ROS. This reduction is indicative of the capacity of both agents to alleviate oxidative damage in FLS, thereby inhibiting their abnormal activation and proliferation—processes known to exacerbate synovial inflammation and cartilage degradation in RA. In parallel, pro-inflammatory cytokines tumor necrosis TNF-α and IL-6, which play central roles in RA immunopathogenesis, were markedly suppressed following pharmacological intervention. Specifically, TNF-α levels decreased from approximately 500 pg/mL in the RA model group to ~300 pg/mL in both treatment groups, representing a reduction of over 30% (Figure 3k). This finding reflects the robust immunomodulatory capacity of both Tofacitinib and NAC in dampening systemic inflammation. The effect on IL-6 expression was even more pronounced: levels in the treatment groups approached those observed in the healthy control group (Figure 3i), indicating highly effective suppression of this cytokine. Given that IL-6 is a critical driver of inflammatory amplification, osteoclast activation, and immune cell recruitment, its reduction further highlights the therapeutic potential of these interventions in modulating the broader cytokine cascade and mitigating RA-associated joint pathology. Taken together, the cytokine and ROS profiles support the conclusion that microneedle-delivered Tofacitinib and NAC effectively suppress key inflammatory and oxidative mediators involved in RA pathogenesis.

### 3.2. Differential Gene Expression

To investigate molecular mechanisms underlying RA pathogenesis and therapeutic intervention, conventional transcriptomic sequencing was employed to analyze differential gene expression across experimental groups relative to healthy controls. The aim was to identify key regulatory pathways in joint tissues involved in inflammatory responses, oxidative stress, and bone/cartilage remodeling. To ensure RNA integrity and the reliability of downstream sequencing data, all biological samples were collected and preserved under rigorously controlled experimental protocols. Following animal euthanasia, right hind limb ankle joints were aseptically dissected under sterile conditions. A meticulous dissection process was immediately carried out to remove skin, muscle, and other non-joint tissues, isolating only articular cartilage and synovial membrane to preserve tissue specificity for transcriptomic analysis. The isolated joint tissues were rapidly immersed in an RNA stabilization reagent, which effectively inhibits RNase activity and preserves the native transcriptional profile. All samples were then stored at –80 °C to maintain molecular stability until total RNA extraction and sequencing were performed. These measures collectively ensured high-quality, tissue-specific RNA samples suitable for reliable differential expression analysis and pathway enrichment studies.

High-throughput RNA sequencing was employed to generate comprehensive whole-transcriptome datasets across all experimental groups. The raw sequencing data underwent rigorous quality control, filtering, and normalization procedures to ensure data reliability and inter-sample comparability. Subsequent differential gene expression analysis identified a set of genes exhibiting statistically significant expression changes between the treatment groups and the RA model group. These differentially expressed genes (DEGs) are considered potential contributors to the molecular mechanisms of RA pathogenesis and therapeutic modulation.

To further investigate the functional relevance of these DEGs, Gene Ontology (GO) enrichment analysis and pathway enrichment analysis were performed. GO terms were categorized into three domains: biological process (BP), cellular component (CC), and molecular function (MF). The GO analysis highlighted biological processes closely related to RA pathology, including inflammatory response, programmed cell death (apoptosis), immune regulation, and bone remodeling. In addition, pathway enrichment analysis revealed significant involvement of several canonical RA-associated signaling pathways, including Janus kinase-signal transducer and activator of transcription (JAK-STAT), tumor necrosis factor (TNF), nuclear factor kappa-light-chain-enhancer of activated B cells (NF-κB), and mitogen-activated protein kinase (MAPK) signaling pathways. These pathways are known to play pivotal roles in cytokine production, immune cell activation, and osteoclast differentiation, thereby contributing to synovial inflammation, joint destruction, and systemic immune dysregulation in RA.

**Figure 4:**
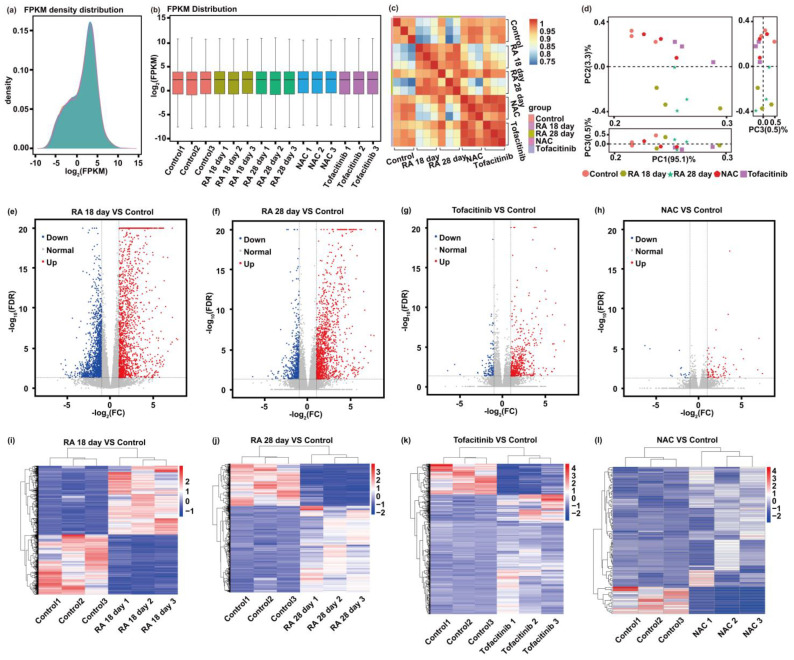
Transcriptomic profiling of RA progression and therapeutic intervention. (**a**) Density distribution of FPKM values across all samples, illustrating global expression patterns of protein−coing genes. The area under each curve equals 1, representing the normalized probability distribution of gene abundance. (**b**) Boxplots of log_2_(FPKM) values across groups, indicating gene expression distribution and sample comparability. (**c**) Sample correlation heatmap showing pairwise expression similarities; higher correlation coefficients denote stronger expression concordance. (**d**) Principal component analysis (PCA) reveals intra−group reproducibility and inter−group transcriptomic divergence. (**e**–**h**) Volcano plots illustrating DEGs between each experimental group and the healthy control: (**e**) RA Day 18 vs. Control, (**f**) RA Day 28 vs. Control, (**g**) Tofacitinib vs. Control, and (**h**) NAC vs. Control. Significantly upregulated and downregulated genes are marked in red and blue, respectively. (**i**–**l**) Heatmaps of DEGs in each comparison group, reflecting transcriptomic alterations under RA pathology and therapeutic intervention: (**i**) RA Day 18, (**j**) RA Day 28, (**k**) Tofacitinib treatment, and (**l**) NAC treatment. Transcriptome sequencing combined with bioinformatics analysis enabled a comprehensive delineation of gene expression patterns associated with RA pathogenesis. In addition to characterizing disease-related transcriptional changes, these data provided mechanistic insights into the molecular effects of Tofacitinib and NAC delivered via microneedlemediated systems. By examining treatment-induced transcriptomic shifts, the study further evaluated how these interventions modulate RA−associated signaling pathways, particularly those involved in inflammatory suppression, immune regulation, and bone remodeling. Collectively, the findings offer new theoretical foundations for precision medicine approaches to RA and support the broader application of microneedle-based drug delivery in chronic inflammatory disease management.

Regarding gene expression abundance (Figure 4a), the FPKM (Fragments Per Kilobase of transcript per Million mapped reads) density distributions across all groups displayed non-normal yet similarly shaped curves, suggesting consistent sequencing depth and uniform data quality across samples—providing a valid basis for downstream differential expression analysis. In addition, the FPKM boxplot (Figure 4b) showed a comparable distribution of gene expression levels among groups, with no evident outliers or technical bias, reinforcing the reliability of intergroup comparisons. To further assess transcriptome similarity, correlation heatmaps and principal component analysis (PCA) were conducted (Figure 4c,d). These analyses revealed that the Tofacitinib and NAC treatment groups clustered more closely with the healthy control group, while the RA 18-day and RA 28-day groups exhibited greater divergence in global gene expression profiles. This result suggests that both treatments partially reversed the RA-induced transcriptomic perturbations, demonstrating therapeutic effects at the systems biology level.

In terms of differentially expressed gene (DEG) distributions and counts (Figure 4e–h), volcano plots provided a visual representation of global transcriptional changes across experimental groups. Both the RA 18-day and RA 28-day model groups exhibited a substantial number of significantly upregulated and downregulated DEGs relative to healthy controls, reflecting widespread transcriptomic dysregulation induced by RA pathogenesis. In contrast, the Tofacitinib and NAC treatment groups displayed markedly fewer DEGs, suggesting that drug interventions mitigated RA-induced gene expression disturbances. Notably, the NAC-treated group showed a dense clustering of DEGs near log_2_(fold change) = 0, indicating minimal transcriptional deviation from baseline and a stronger restorative effect on gene expression patterns. Among all experimental conditions, the NAC group exhibited the lowest total number of DEGs, both in upregulated and downregulated categories, underscoring its potential as a more effective modulator of global gene expression in the RA model. Further confirmation was obtained through hierarchical clustering heatmaps (Figure 4i–l), which illustrated inter-sample gene expression similarities. In the RA model groups, DEGs formed distinct clusters, diverging significantly from the healthy control group, indicative of disease-driven transcriptomic remodeling. Conversely, the Tofacitinib and NAC treatment groups clustered more closely with the healthy controls, reinforcing the observation that both interventions partially restored gene expression homeostasis and attenuated RA-associated transcriptional dysregulation.

### 3.3. GO Biological Process Analysis

Building upon the preceding DEG analysis, Gene Ontology (GO) enrichment analysis was performed to systematically investigate differences in molecular function (MF), cellular component (CC), and biological process (BP) between the experimental groups and healthy controls. This approach aimed to uncover key molecular mechanisms underlying RA pathogenesis and to assess how pharmacological interventions modulate these pathways at the functional level. Comparative GO profiling was conducted for each of the four experimental groups—Tofacitinib-treated, NAC-treated, RA 18-day, and RA 28-day—against the healthy control group. Enrichment significance was evaluated using adjusted *p*-values, and the resulting functional categories were ranked based on statistical relevance. To visually interpret the regulatory trends and functional activation states across conditions, GO term-based heatmaps were generated, providing a comparative overview of biological pathway enrichment and expression directionality among the groups.

GO enrichment analysis revealed pronounced functional alterations in the RA 18-day and RA 28-day groups compared to healthy controls, indicating substantial transcriptional reprogramming across different stages of disease progression. Among the enriched terms, inflammatory response, immune response, and immune system processes were markedly overrepresented, suggesting sustained immunopathological activation in RA. Chemokine-mediated signaling pathways exhibited persistent and robust enrichment, underscoring their pivotal role in driving leukocyte recruitment, synovial infiltration, and the amplification of local inflammation. Additionally, genes involved in both innate and adaptive immune responses were significantly enriched, reflecting the synergistic contribution of multiple immune compartments—including macrophages, T cells, and dendritic cells—to RA pathogenesis. These findings support the notion that RA involves a complex, stage-specific immune network, wherein both early innate triggers and later adaptive cascades orchestrate the chronic inflammatory milieu.

**Figure 5:**
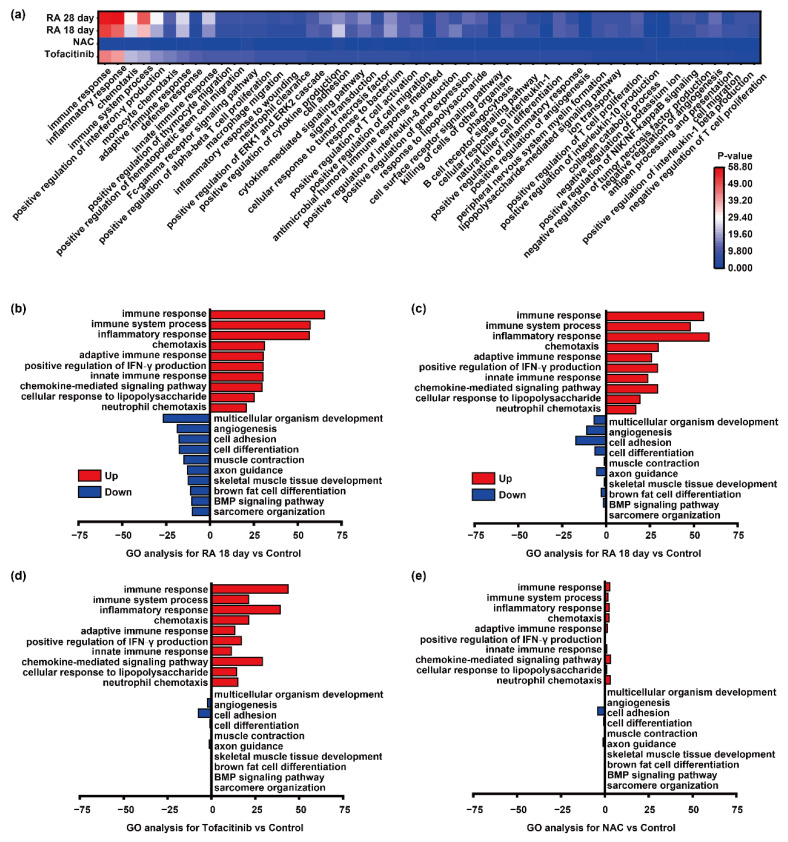
Functional enrichment analysis of DEGs across RA pathology and treatment groups based on GO biological processes. (**a**) Heatmap summarizing the enrichment significance (*p*-values) of GO biological processes among the RA Day 18, RA Day 28, Tofacitinib-treated, and NAC-treated groups relative to the healthy control group. Each column represents a group, and each row denotes a significantly enriched GO term. Warmer colors indicate higher statistical significance. (**b**–**e**) Bar plots illustrating the direction and magnitude of GO term enrichment in: (**b**) RA Day 18 vs. Control; (**c**) RA Day 28 vs. Control; (**d**) Tofacitinib-treated vs. Control; (**e**) NAC-treated vs. Control. Red bars indicate upregulated biological processes, while blue bars represent downregulated ones. Across the RA progression and treatment comparisons, upregulated GO terms predominantly involve immune response, innate/adaptive immune activation, inflammatory cytokine production (e.g., IFN-γ, chemokine signaling), and cellular responses to bacterial components (e.g., lipopolysaccharide). In contrast, downregulated processes are mainly related to musculoskeletal development, such as muscle contraction, cell adhesion, sarcomere organization, and axon guidance. Treatment with Tofacitinib or NAC resulted in partial reversal of these immuno-inflammatory enrichments and promoted restoration of developmental and tissue organization pathways, indicating therapeutic modulation at the transcriptomic level.

Further GO analyses revealed significant enrichment of biological processes related to signal transduction, cell adhesion, and chemotaxis in the RA model groups. Notably, pathways such as neutrophil chemotaxis and general cell chemotaxis were prominently upregulated, suggesting their key involvement in establishing a pro-inflammatory microenvironment within the synovial tissue. Concurrently, the positive regulation of tumor necrosis factor (TNF) production and modulation of cytosolic calcium ion concentration were enhanced, indicating that aberrant activation of inflammatory signaling may promote pro-inflammatory cytokine release and immune cell recruitment via calcium-mediated signaling cascades (Figure 5a). At the cytokine level, interferon-γ production and cytokine-mediated signaling pathways were significantly elevated, underscoring the central role of pro-inflammatory mediators in RA progression. Dysregulated expression of TNF and members of the interleukin (IL) family likely contributed to synovial hyperplasia, osteoclast activation, and cartilage matrix degradation. Moreover, heightened cellular responses to lipopolysaccharide (LPS) and interferon-γ imply the presence of a synergistic amplification loop between pathogen-associated molecular patterns (PAMPs) and inflammatory cytokines, further exacerbating the disease state. Importantly, treatment with Tofacitinib and NAC substantially reduced the enrichment of RA-associated inflammatory and immune-related GO terms. This suggests that both agents may attenuate immune cell activation and pro-inflammatory cytokine release by modulating key signaling pathways, including JAK-STAT, TNF, and MAPK. These regulatory effects likely contribute to reduced tissue inflammation and structural damage. Taken together, these findings not only elucidate the molecular basis of the therapeutic effects observed with Tofacitinib and NAC, but also underscore the potential of microneedle-based drug delivery systems as an effective strategy for modulating immune responses and mitigating RA progression. The in-depth interpretation of GO enrichment results provides valuable insights into the complex immunopathology of RA and offers a theoretical foundation for developing targeted and precision-guided interventions.

In the heatmaps, red and blue indicate upregulated and downregulated biological process terms, respectively. In the RA 18 day and RA 28 day groups (Figure 5b,c), a substantial number of immune system-related biological processes were markedly upregulated, including immune response, immune system process, inflammatory response, and chemotaxis, reflecting robust immune activation and progressive inflammatory pathology. Notably, the positive regulation of interferon-γ production and the enrichment of chemokine-mediated signaling pathways further underscored enhanced immune cell recruitment and sustained inflammatory signaling. In particular, the upregulation of response to lipopolysaccharide and neutrophil chemotaxis indicates an intensified innate immune response, likely contributing to amplified inflammation and synovial infiltration in the RA model.

In contrast, downregulated GO terms were predominantly associated with tissue development, repair, and structural maintenance. These included multicellular organism development, angiogenesis, cell adhesion, and cell differentiation, implying that chronic inflammation compromises tissue regeneration and remodeling capacity. Moreover, suppressed biological processes such as muscle contraction, axon guidance, and skeletal muscle tissue development suggest potential structural and functional damage to muscular tissues. The downregulation of brown adipocyte differentiation and BMP signaling pathways may reflect impaired energy metabolism and cell growth regulation under inflammatory conditions. Additionally, reduced activity in sarcomere organization regulation indicates disruption of muscle structural integrity, which may further exacerbate joint dysfunction and mobility impairment.

Notably, Gene Ontology (GO) differential analysis between the Tofacitinib and NAC intervention groups and the RA 18-day and 28-day model groups revealed a marked reduction in the upregulation of immune- and inflammation-related pathways, accompanied by an overall narrowing of GO term disparities (Figure 5d,e). These findings indicate that both Tofacitinib and NAC effectively suppress immune hyperactivation and attenuate aberrant enrichment of inflammatory signaling pathways in the RA model. Specifically, terms related to immune response, inflammatory response, and chemokine-mediated pathways were significantly downregulated. Meanwhile, suppression of tissue repair-associated processes—such as cell differentiation, angiogenesis, and muscle development—was partially reversed following drug intervention. This suggests that Tofacitinib and NAC not only mitigate inflammation but also contribute to the restoration of tissue repair and regeneration capacity.

In summary, GO enrichment analysis comprehensively delineated the pathological landscape of RA progression, characterized by persistent immune activation and broad inhibition of developmental and metabolic processes. Treatment with Tofacitinib and NAC partially corrected this imbalance by downregulating immune and inflammatory responses and restoring key biological functions related to tissue structure and metabolism. These results provide robust molecular evidence supporting the anti-inflammatory and tissue-protective effects of Tofacitinib and NAC, and highlight their therapeutic potential for RA and other autoimmune inflammatory disorders.

### 3.4. Pathway Analysis

In transcriptomic research, pathway analysis serves as a critical tool for functional annotation, enabling the identification of enrichment patterns of DEGs within specific biological signaling pathways. This approach facilitates the elucidation of key molecular mechanisms underlying disease pathogenesis. Distinct from GO analysis—which primarily categorizes gene functions—pathway analysis focuses on the interconnected roles and cooperative interactions of genes within complex biological networks, including signal transduction, metabolic regulation, and immune responses. For multifactorial diseases such as RA, which involve dysregulated immune and inflammatory signaling, reliance solely on DEG screening and GO enrichment provides limited mechanistic insight. In contrast, pathway analysis offers a more integrative perspective by mapping DEGs onto known regulatory cascades, thereby uncovering the core signaling pathways aberrantly activated during RA progression and clarifying the mechanistic basis of pharmacological interventions. Thus, pathway analysis is indispensable for a comprehensive understanding of transcriptomic dysregulation and drug response in RA models.

**Figure 6:**
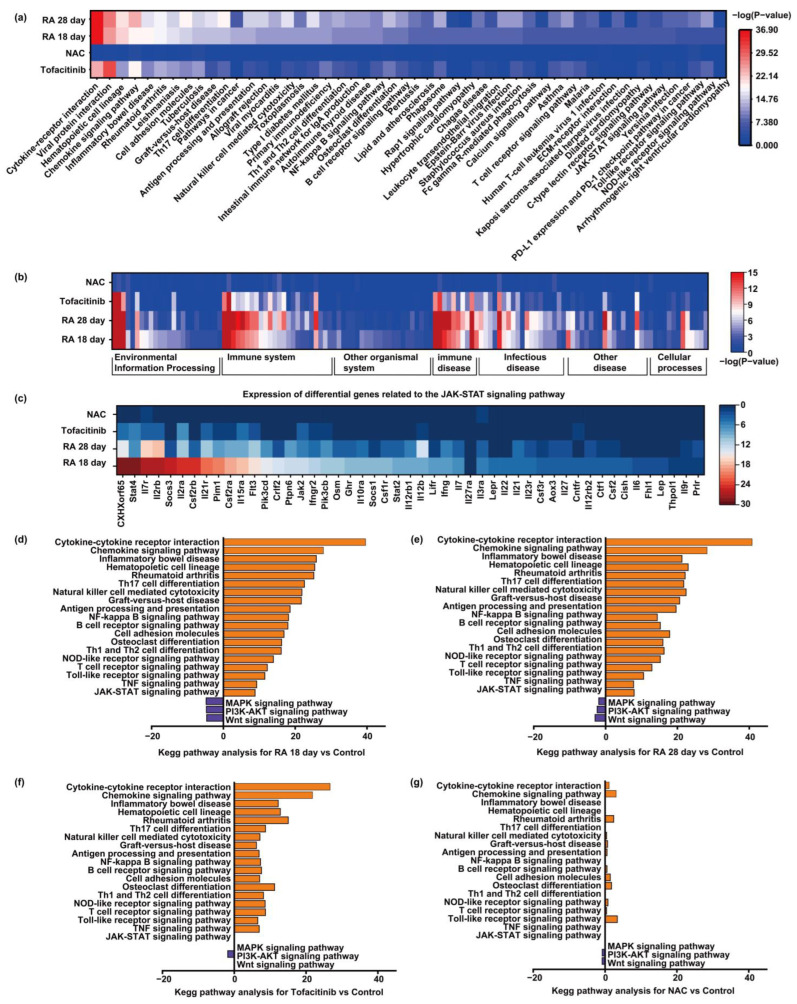
KEGG signaling pathway enrichment analysis across RA pathological and treatment groups. (**a**) Heatmap showing the −log_10_(*p*-value) of significantly enriched KEGG signaling pathways among the RA Day 18, RA Day 28, Tofacitinib-treated, and NAC-treated groups compared to the healthy control group. Notable pathways include JAK-STAT signaling, MAPK signaling, PI3K-Akt pathway, and multiple immune and cytokine-mediated pathways. (**b**) Classification of differentially enriched pathways into functional categories including: Environmental Information Processing, Cellular Processes, Organismal Systems, Immune System, Immune-Related Diseases, Infectious Diseases, and Other Human Diseases. The RA and treatment groups display distinct enrichment patterns across these categories, reflecting disease progression and therapeutic modulation. (**c**) Heatmap displaying the expression levels of key genes involved in the JAK-STAT signaling pathway across all groups. Genes such as CXCR4, IL6, IL1β, IFNγ, STAT1/3, and SOCS3 exhibit significant upregulation in RA groups, with partial normalization following Tofacitinib or NAC treatment. (**d**–**g**) Bar plots illustrating the enrichment scores of KEGG signaling pathways in: (**d**) RA Day 18 vs. Control, (**e**) RA Day 28 vs. Control, (**f**) Tofacitinib vs. Control, (**g**) NAC vs. Control. Orange bars represent upregulated signaling pathways, while blue bars denote downregulated ones. Consistently enriched upregulated pathways include cytokine-cytokine receptor interaction, chemokine signaling, Th1/Th2/Th17 cell differentiation, NF-κB, and Toll-like receptor signaling—hallmarks of RA inflammatory pathology. Both Tofacitinib and NAC treatments downregulated key proinflammatory and immune-related pathways (e.g., JAK-STAT and MAPK), suggesting distinct but overlapping immunomodulatory effects at the transcriptomic level.

Building upon these considerations, the present study conducted a systematic pathway enrichment analysis comparing four experimental groups—RA day 18, RA day 28, Tofacitinib intervention, and NAC intervention—with healthy controls. To visualize the transcriptomic alterations associated with disease progression and pharmacological treatment, multiple comparative analyses were performed, including differential pathway heatmaps, functional classification heatmaps, and bar plots of significantly upregulated and downregulated pathways. Figure 6a presents a differential pathway heatmap displaying variations in pathway activity across groups, with corresponding *p* values indicating statistical significance. This approach enables an intuitive, comparative overview of molecular pathway dynamics during RA progression and following therapeutic intervention.

Pathway analysis revealed marked alterations in several key signaling cascades in both the RA 18-day and 28-day model groups relative to healthy controls. These included pathways implicated in inflammatory responses, immune dysregulation, and bone degradation. Notably, the cytokine–cytokine receptor interaction pathway, a pivotal initiator of inflammatory signaling cascades, was significantly enriched. This pathway governs the expression and release of critical pro-inflammatory mediators such as TNF-α, IL-6, and IFN-γ, and is recognized as a central driver of chronic inflammation in RA. In addition, the chemokine signaling pathway, Toll-like receptor (TLR) pathway, and NOD-like receptor pathway—all essential components of innate immunity—were markedly upregulated. These pathways mediate the recruitment and activation of immune cells and are instrumental in amplifying local inflammation in synovial tissues. Their enrichment underscores the persistent activation of innate immune responses in RA, contributing to ongoing immune stimulation and chronic joint inflammation.

Meanwhile, the upregulation of the JAK–STAT signaling pathway suggests aberrant activation of adaptive immune responses in the RA model. As a central signaling cascade downstream of various cytokines, the JAK–STAT pathway regulates T cell activation, B cell differentiation, and the transcription of multiple pro-inflammatory mediators. Its activation is tightly associated with Th1/Th2 and Th17 cell differentiation, indicating a dysregulated helper T cell subset balance in RA pathology. Furthermore, the T cell receptor (TCR) and B cell receptor (BCR) signaling pathways were significantly activated in both the RA 18-day and 28-day groups, reflecting persistent immune surveillance and exacerbated autoimmune responses that contribute to synovitis and cartilage destruction. A deeper analysis of JAK–STAT pathway–related genes revealed robust upregulation of Csf2rb, Il2ra, Il2rb, and Il7r, as well as downstream signaling molecules such as Stat4, Socs3, and CXHXorf65. These changes were most pronounced at day 18 post-induction, indicating early-stage hyperactivation of JAK–STAT signaling. Moreover, a broad range of inflammatory cytokines and receptors—Il6, Il12rb2, Il23r, Ifng, Osm, Il21, and Il22—were also significantly upregulated in the RA groups, reflecting a sustained and complex cytokine network that promotes immune dysregulation and joint pathology. In contrast, the combination treatment with Tofacitinib and NAC markedly attenuated these gene expression abnormalities. Most of the aforementioned gene expression levels were substantially reduced, approaching those observed in healthy controls. This finding suggests that microneedle-mediated delivery of Tofacitinib and NAC effectively suppresses hyperactive JAK–STAT signaling, thereby modulating adaptive immune responses, reducing inflammatory burden, and mitigating tissue damage in RA.

Furthermore, the NF-κB and MAPK signaling pathways—two classical regulators of inflammation—were notably enriched in both the RA 18-day and RA 28-day groups. These pathways play pivotal roles in promoting the expression of inflammatory cytokines, synoviocyte proliferation, and immune cell activation, and their sustained activation further confirms the presence of chronic inflammatory responses during RA progression. In addition to immune and inflammatory signaling, aberrant activation of bone destruction–related pathways emerged as another hallmark of RA pathology. Specifically, enrichment of the osteoclast differentiation pathway in RA model groups suggests heightened osteoclastogenesis, which may accelerate cartilage erosion and bone tissue degradation. Conversely, both Tofacitinib and NAC interventions significantly reduced the activation levels of these pro-inflammatory and bone-destructive pathways. This finding indicates that microneedle-mediated drug delivery can attenuate pathological signaling cascades and potentially suppress the progression of joint damage. Notably, the Tofacitinib group exhibited a marked reduction in JAK–STAT pathway activity, consistent with its known pharmacological mechanism as a JAK inhibitor, which effectively inhibits cytokine-mediated signal transduction, thereby alleviating synovitis and joint deterioration in RA. Furthermore, both treatments led to substantial downregulation of T cell receptor and B cell receptor signaling pathways, suggesting their ability to modulate adaptive immune dysregulation associated with RA. Regarding the osteoclast differentiation pathway, its decreased enrichment following treatment implies suppression of excessive osteoclast formation, potentially contributing to slower bone degradation and improved structural joint integrity.

To systematically characterize the functional classifications of signaling pathways implicated in the RA model, this study further analyzed pathway classification results across the four experimental groups relative to healthy controls (Figure 6b). The enriched pathways were primarily categorized into environmental information processing, cellular processes, organ systems, immune system, immune-related diseases, infectious diseases, and human diseases. Among these, the immune system, immune-related diseases, and infectious diseases categories exhibited the most pronounced alterations in the RA 18-day and RA 28-day groups. This pattern reflects extensive immune dysregulation during RA progression, characterized by concurrent activation of both innate and adaptive immune responses. Furthermore, the upregulation of pathways associated with infectious diseases suggests that the RA model may trigger infection-mimicking immune activation, which may underlie the persistence of chronic inflammation in RA. These classification results are consistent with individual pathway enrichment findings and collectively reinforce the notion that RA is a systemic autoimmune disorder rather than a disease confined to local joint pathology. By contrast, the Tofacitinib and NAC intervention groups demonstrated markedly reduced enrichment of pathways within the immune system, immune-related diseases, and infectious diseases categories. Notably, key pro-inflammatory pathways such as JAK-STAT and TNF signaling were significantly downregulated. These results indicate that both interventions effectively restore immune homeostasis, suppress pro-inflammatory cytokine signaling, and attenuate immune cell hyperactivation. Moreover, the observed downregulation of infection-like response pathways suggests that these treatments may also mitigate non-specific immune overactivation, thereby exerting broad-spectrum control over RA-associated chronic inflammation. Overall, these findings substantiate the mechanistic rationale for employing Tofacitinib and NAC as therapeutic strategies in RA and underscore their clinical potential in modulating dysregulated immune and inflammatory networks.

To elucidate the dynamic expression patterns of key signaling pathways across experimental conditions, bar plots were generated to visualize the top upregulated and downregulated pathways in each group (Figure 6c–f). Pathways were classified by regulation direction—upregulated (orange) and downregulated (blue)—highlighting the transcriptional shifts during RA progression and treatment.

In the RA 18-day and RA 28-day groups, numerous upregulated pathways were predominantly associated with inflammatory responses and immune activation, including cytokine–cytokine receptor interaction, chemokine signaling, NF-κB signaling, TNF signaling, Th1/Th2 and Th17 cell differentiation, as well as Toll-like and NOD-like receptor signaling. Collectively, these pathways form a core network of hyperactivated innate and adaptive immune responses that drive chronic inflammation in RA. The concurrent upregulation of T cell receptor and B cell receptor signaling pathways further indicated heightened lymphocyte activation, corroborated by enrichment of antigen processing and presentation and natural killer cell-mediated cytotoxicity pathways. Additionally, pathways related to autoimmune diseases, such as RA and inflammatory bowel disease, were significantly upregulated, underscoring the systemic immune dysregulation characteristic of RA. Conversely, pathways involved in cell survival and tissue repair, including MAPK, PI3K-AKT, and Wnt signaling, were downregulated in the RA groups. This suggests that the pro-inflammatory microenvironment may suppress regenerative signaling, impairing joint tissue homeostasis and repair capacity. Importantly, these aberrant enrichments were substantially attenuated in the Tofacitinib and NAC intervention groups. Inflammation- and immune-related pathway activity was notably reduced, while repair- and survival-associated pathways showed varying degrees of restoration. Notably, the JAK-STAT pathway exhibited significant downregulation in the Tofacitinib group, aligning with its pharmacological role as a JAK inhibitor. Furthermore, pathways related to T helper cell differentiation, T/B cell activation, and innate immune responses trended toward normalization post-intervention, indicating that both treatments not only suppress pro-inflammatory signals but also enhance immune regulation and facilitate tissue repair—together contributing to a comprehensive therapeutic effect.

### 3.5. Bioinformatics Association of Pathological Features and Gene Expression Differences

**Figure 7:**
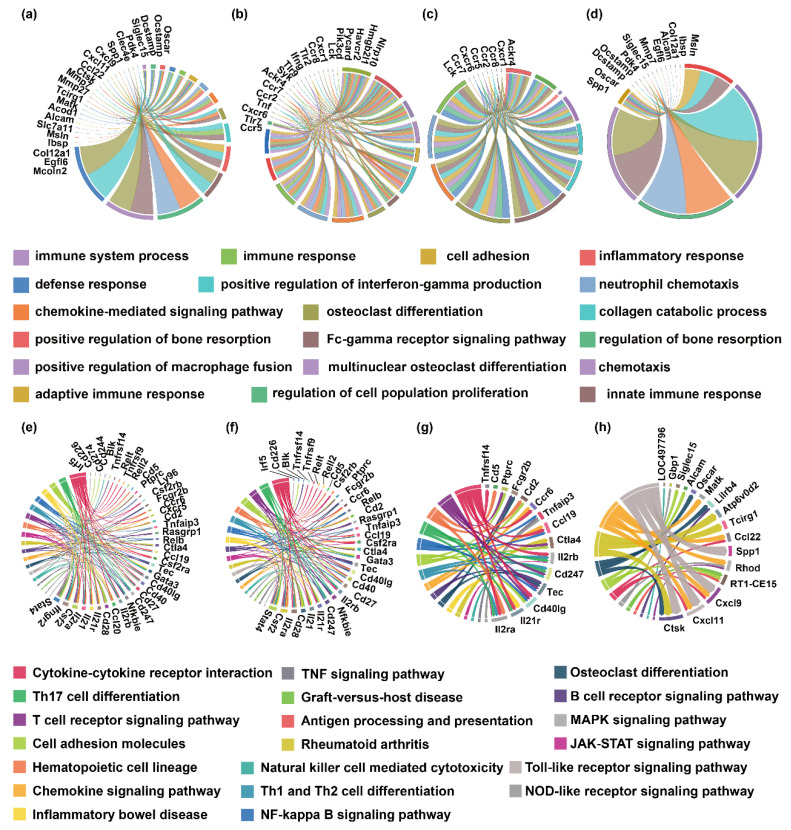
Chord diagrams revealing gene–function associations based on GO and KEGG enrichment analyses in the RA model and treatment groups. Chord plots depict the relationships between differentially expressed genes and their corresponding biological processes or signaling pathways, with colored arcs representing specific GO terms (**a**–**d**) or KEGG pathways (**e**–**h**). Each chord illustrates a gene enriched in one or more functional categories, highlighting the multifaceted roles of hub genes in RA pathogenesis and therapeutic response. GO enrichment-based diagrams: (**a**) RA Day 18 group vs. healthy control, showing strong associations between immune system processes, cell adhesion, chemotaxis, and osteoclast differentiation. (**b**) RA Day 28 group vs. healthy control, indicating expanded functional engagement in defense response, bone resorption, and macrophage fusion. (**c**) Tofacitinib-treated group vs. healthy control, showing a distinct shift in GO term associations with marked reduction in immune and inflammatory signatures. (**d**) NAC-treated group vs. healthy control, revealing more restricted GO engagement, yet retaining some immune-related functions. KEGG pathway-based diagrams: (**e**) RA Day 18 group vs. healthy control, with key genes enriched in cytokine-cytokine receptor interaction, chemokine signaling, and NF-κB pathways. (**f**) RA Day 28 group vs. healthy control, reflecting continued enrichment in pro-inflammatory signaling such as Th17 differentiation and Toll-like receptor signaling. (**g**) Tofacitinib-treated group vs. healthy control, showing extensive suppression of immune-related signaling, particularly JAK-STAT and MAPK pathways. (**h**) NAC-treated group vs. healthy control, with moderate attenuation of inflammatory pathway activity, including JAK-STAT and B cell receptor signaling.

To further elucidate the functional roles of key genes in the pathogenesis of RA, this study constructed chord diagrams illustrating the associations between genes and GO terms or signaling pathways (Figure 7). These visualizations provide a systematic integration of gene-function relationships across multiple biological dimensions, enabling the identification of central regulatory genes involved in immune and inflammatory networks. In the gene-GO association analysis, several GO terms closely related to immune and inflammatory responses—including immune response, inflammatory response, and immune system process—were significantly enriched. A subset of key associated genes was identified, including Ccr5, Tlr7, Cxcr6, Tnf, Ccr2, Ccr7, Ackr4, Syk, Tlr9, Ifng, Tlr2, Ccr8, and Cxcr1. These genes are critically involved in the regulation of inflammatory mediators, chemokine signaling, and innate and adaptive immunity. Specifically, Ccr5, Ccr2, Ccr7, and Cxcr6 function in the chemokine signaling pathway, facilitating immune cell recruitment and migration. Tlr2, Tlr7, and Tlr9 are members of the Toll-like receptor (TLR) family, essential for pathogen recognition and innate immune activation. Tnf and Ifng, as classical pro-inflammatory cytokines, contribute to the amplification of inflammatory signaling and the modulation of immune responses, highlighting their central roles in RA pathophysiology.

Further analysis revealed that genes such as Ccr5, Cxcr6, Ccr2, Ccr7, Ackr4, and Cxcr1 were highly enriched in GO terms associated with chemotaxis, including monocyte chemotaxis, macrophage migration, and thymocyte migration. These findings suggest that active immune cell trafficking within the inflamed synovial microenvironment is a key driver of synovial inflammation and tissue damage in RA. Importantly, the expression levels of these chemotaxis-related genes were markedly reduced in the Tofacitinib and NAC intervention groups compared to the RA 18-day and RA 28-day groups (Figure 7a,b). This indicates that both interventions may alleviate pathological immune cell recruitment and activation by inhibiting chemokine-mediated signaling and the release of pro-inflammatory mediators, thereby mitigating synovial inflammation and tissue destruction. Collectively, these results highlight the therapeutic potential of Tofacitinib, a JAK-STAT pathway inhibitor, and NAC, an antioxidant agent, in modulating aberrant immune responses and suppressing inflammatory cell infiltration in RA.

Figure 7e–h depict chord diagrams illustrating the associations between genes and immune-related signaling pathways across the RA model groups, highlighting the functional involvement and activity levels of key genes within critical inflammatory networks. The analysis revealed significant enrichment of pathways such as cytokine–cytokine receptor interaction, JAK-STAT, Toll-like receptor, T cell receptor, and NF-κB signaling, implicating pivotal genes including Il2rb, Il2ra, Stat4, and Ifngr2. These genes are extensively involved in immune signal recognition, transduction, and downstream effector responses, underscoring their central roles in regulating inflammation and mediating immune dysregulation in RA pathogenesis. On day 18 post-induction, the RA model exhibited pronounced upregulation of pro-inflammatory cytokines Ifng, Tnf, and Il1b, which were notably enriched in the NF-κB and NOD-like receptor signaling pathways, reflecting a robust early inflammatory state. Concurrently, early activation of the T cell receptor pathway was evidenced by increased expression of Lck and PI3K subunits, suggesting heightened T cell responsiveness. By day 28, the inflammatory cytokine profile shifted, with immune signaling becoming more oriented toward regulatory mechanisms. Notably, elevated expression of Il2ra and Il21r contributed to the activation of the JAK-STAT pathway and promotion of Th17 cell differentiation, a key event in RA progression. In parallel, the upregulation of Cd28 and Ctla4 pointed to a more complex immunological landscape, indicating a dynamic interplay between T cell activation and inhibitory signaling as the disease progressed.

The intervention groups demonstrated distinct regulatory profiles. Tofacitinib effectively downregulated the expression of Il2ra and Il21r, thereby suppressing JAK-STAT pathway activity and upregulating Ctla4, which may contribute to the attenuation of T cell-mediated inflammatory responses. However, its modulatory effect on the osteoclast differentiation pathway appeared limited. In contrast, N-acetylcysteine (NAC) exhibited a weaker impact on adaptive immune regulation, yet significantly reduced the expression of bone resorption–associated genes, including Ctsk and Oscar, suggesting a potential role in inhibiting osteoclast-mediated bone destruction. Additionally, NAC suppressed the expression of chemokines such as CXCL9 and CXCL11, potentially limiting the recruitment of inflammatory cells into affected tissues. These findings underscore the complementary mechanisms of action between Tofacitinib and NAC: while Tofacitinib primarily targets T cell–driven signaling pathways, NAC exhibits advantages in modulating bone metabolism and oxidative stress–associated inflammatory responses. Their combined application may thus provide synergistic benefits, simultaneously correcting immune dysregulation and preventing progressive joint damage.

Further investigation is carried out to explore whether Il2rb and Ctsk expression levels could serve as predictive biomarkers for treatment responsiveness and therapeutic efficacy, potentially enabling personalized intervention strategies in RA. Overall, the data suggest that distinct signaling pathways dominate at different stages of RA progression, emphasizing the importance of phase-specific therapeutic targeting. Early intervention strategies may benefit from the suppression of acute-phase cytokines such as Ifng and Tnf, while modulation of Il2ra, Il21r, and Th17 differentiation–associated genes may be more effective in controlling chronic inflammation and immune dysregulation during later disease stages.

**Figure 8:**
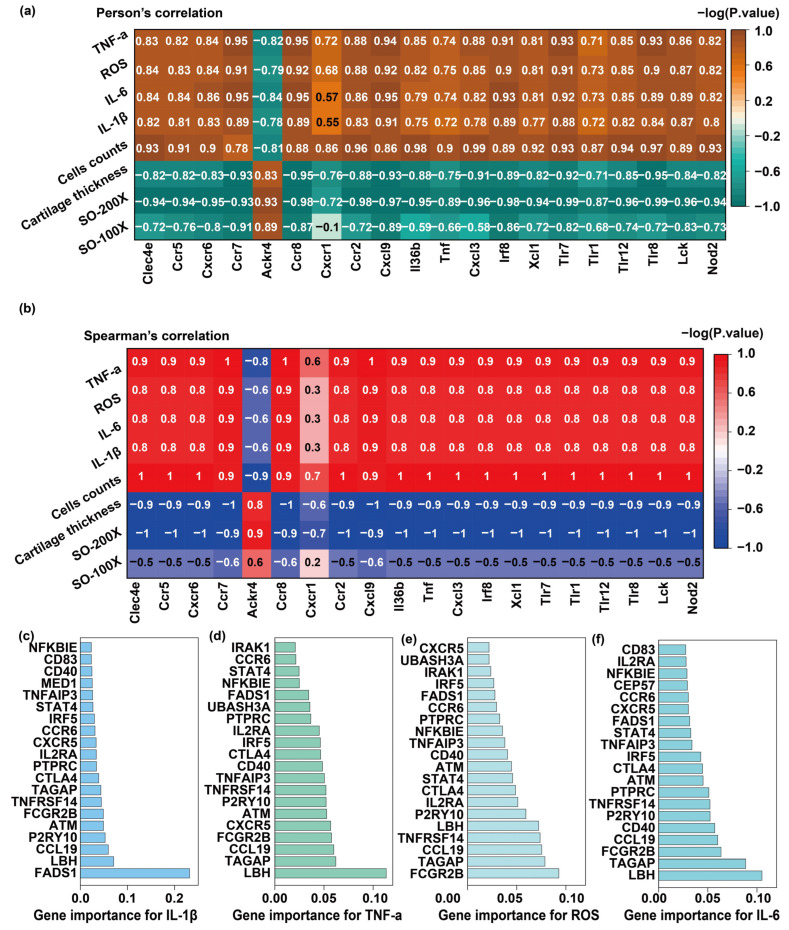
Comprehensive correlation and machine learning-based feature importance analysis reveals associations between RA-related gene expression and pathological indicators. (**a**) Pearson correlation heatmap displaying linear associations between gene expression profiles and eight key pathological features across five experimental groups: healthy control, RA Day 18, RA Day 28, Tofacitinib-treated, and NAC-treated groups. Pathological features include pro-inflammatory cytokines (TNF-α, IL-6, IL-1β), oxidative stress marker (ROS), synovial immune cell infiltration (cell counts), cartilage degradation index (cartilage thickness), and histological scores from Safranin O staining (SO-200×, SO-100×). Positive correlations are represented by warmer colors, while negative correlations are indicated by cooler shades. (**b**) Spearman correlation heatmap evaluating the rank-based, non-linear associations between the same pathological indicators and gene expression across all experimental conditions. This analysis highlights robust monotonic trends, with strong positive correlations observed between inflammatory gene signatures and elevated TNF-α, IL-6, ROS, and IL-1β levels, whereas cartilage thickness shows consistent negative correlation with pro-inflammatory markers and immune cell-related genes (e.g., Cxcl9, Tlr1, Tlr7, Irf8, Lck, Nod2), suggesting their potential roles in joint tissue degeneration. (**c**) Random forest-based feature importance analysis identifying the top-ranked genes contributing to IL-1β expression across the dataset. Genes such as NFKBIE, CD83, TNFAIP3, STAT4, and FADS1 show high importance scores, indicating potential regulatory influence on IL-1β production and RA-associated inflammation. (**d**) Feature importance analysis for TNF-α expression, highlighting key genes involved in its regulation. Notably, IRAK1, FADS1, STAT4, IL2RA, and CTLA4 rank among the most influential features, implicating both innate immunity and T cell co-stimulatory signaling in TNF-α-mediated pathogenesis. (**e**) Gene importance scores for ROS (reactive oxygen species) levels, identifying genes such as CXCR5, IRF5, ATM, CD40, TAGAP, and FCGR2B as major contributors. These genes are associated with immune activation, redox regulation, and apoptotic signaling, suggesting their relevance to oxidative stress and joint damage in RA. (**f**) Feature importance for IL-6 expression, with genes including CD83, IL2RA, CEP57, TNFRSF14, and CXCR5 showing high predictive contribution. The identified gene set spans adaptive immune pathways, transcriptional regulators, and cytokine signaling, offering insights into mechanisms of IL-6 dysregulation. This figure integrates correlation metrics and machine learning-based variable importance to uncover candidate regulatory genes that may serve as potential biomarkers or therapeutic targets in RA progression and treatment response.

Correlation analyses revealed strong positive associations between key genes and pro-inflammatory cytokines IL-1β, IL-6, and TNF-α. Notably, genes such as Clec4e, Ccr5, Cxcr6, Ccr7, Cxcl9, Tlr7, and Nod2 exhibited high correlation coefficients (r = 0.8–1.0) with these cytokines (Figure 8a), indicating that their elevated expression is closely linked to heightened inflammatory responses and may play a critical role in driving RA pathogenesis. These genes are predominantly involved in chemokine signaling, Toll-like receptor signaling, and inflammatory cytokine regulation pathways, suggesting that they may promote the release of inflammatory mediators by activating downstream cascades such as NF-κB and JAK-STAT. This, in turn, could enhance immune cell recruitment, amplify local inflammatory responses, and exacerbate synovial inflammation and joint damage in RA.

Spearman correlation analysis was performed to evaluate monotonic associations between gene expression and clinical parameters, using correlation coefficients ranging from −1 to 1 (Figure 8b). A positive coefficient (>0) indicates that an increase in one variable corresponds to an increase in the other, whereas a negative coefficient (<0) suggests an inverse relationship. Coefficients near zero imply little to no correlation. In this study, the clinical indicators analyzed included cartilage thickness, osteoclast counts, levels of inflammatory cytokines (IL-1β, IL-6, TNF-α), and reactive oxygen species (ROS). The gene set encompassed chemokine receptors (e.g., Ccr5, Cxcr6), Toll-like receptors (e.g., Tlr7, Tlr8), and other inflammation-related genes. The correlation results revealed notable associations between specific gene expression patterns and disease-related phenotypes, providing important mechanistic insights into the pathogenesis of RA. These findings suggest that certain genes may serve as molecular indicators of inflammation severity, immune activation, or tissue degradation, and may offer potential targets for therapeutic intervention or biomarker development.

Specifically, cartilage thickness exhibited a strong negative correlation with the expression levels of several key genes, including Ccr7, Ccr8, Cxcl9, Tlr7, and Nod2, with correlation coefficients approaching −1. This trend suggests that elevated expression of these genes is closely associated with cartilage degradation in RA. A potential mechanism underlying this association is the activation of pro-inflammatory signaling pathways by these genes, which may induce the expression of cartilage-degrading enzymes, such as matrix metalloproteinases (MMPs), leading to progressive breakdown of the cartilage extracellular matrix. In RA, dysregulated immune responses and chronic inflammation sustain high expression of inflammatory mediators and immune-related genes, which can exacerbate joint tissue damage and accelerate disease progression. These findings highlight the potential of Ccr7, Tlr7, and related genes as molecular markers of cartilage destruction and suggest their possible utility as therapeutic targets for preserving joint integrity in RA.

A strong positive correlation was also observed between increased osteoclast counts and the elevated expression of inflammation-related genes. Notably, Clec4e, Ccr5, Cxcr6, Cxcl9, Tlr7, and Nod2 exhibited correlation coefficients approaching 1, suggesting that their upregulation may be closely linked to inflammatory cell infiltration and osteoclast activation in bone tissues of RA models. These genes likely contribute to the promotion of osteoclast differentiation and functional activation, thereby enhancing bone resorption and exacerbating bone erosion commonly seen in RA.

Furthermore, the strong positive correlation between reactive oxygen species (ROS) levels and pro-inflammatory gene expression further underscores the interplay between oxidative stress and inflammatory processes in RA pathogenesis. Excessive ROS generation is known to cause mitochondrial dysfunction, cellular apoptosis, and tissue damage, and can potentiate inflammation through the activation of NF-κB and MAPK signaling pathways. These findings highlight that targeting ROS may not only mitigate oxidative damage but also suppress inflammation, positioning ROS regulation as a critical therapeutic strategy for RA management.

Notably, Ackr4 expression exhibited a negative correlation with key inflammatory cytokines IL-1β, IL-6, and TNF-α, with correlation coefficients ranging from −0.6 to −0.8, while displaying a strong positive correlation (coefficient ~0.8) with cartilage thickness. These findings suggest that Ackr4 may play a protective role in the pathogenesis of RA. As an atypical chemokine receptor, Ackr4 is thought to modulate chemokine gradients and reduce the local recruitment of inflammatory cells, thereby alleviating synovial inflammation and slowing cartilage degradation. This implicates Ackr4 as a potential therapeutic target for RA intervention.

Collectively, the correlation analysis underscores that multiple pro-inflammatory genes—including Clec4e, Ccr5, Cxcr6, Cxcl9, Tlr7, and Nod2—are strongly associated with elevated inflammatory cytokine levels, ROS accumulation, and bone destruction in RA. These genes likely exacerbate disease progression by amplifying inflammatory signaling and promoting oxidative stress-mediated tissue damage. In contrast, genes such as Ackr4 may counteract these pathogenic processes and confer anti-inflammatory and tissue-protective effects. These insights highlight the dual regulatory landscape of RA, involving both pro-inflammatory drivers and protective modulators, and point to the need for precision-targeted therapies. Future studies should further delineate the mechanistic roles of these key genes and evaluate their potential as biomarkers or targets for combination therapeutic strategies, aiming to restore immune homeostasis and prevent joint destruction in RA.

In RA research, elucidating the relationships between gene expression and inflammatory mediators is essential for unraveling disease pathogenesis. Leveraging machine learning approaches, particularly ensemble methods, provides a powerful framework to identify key regulatory genes from high-dimensional and complex transcriptomic datasets. In this study, a random forest model was employed to assess the contribution of RA-associated genes to the levels of TNF-α, IL-6, IL-1β, and reactive oxygen species (ROS), with the goal of identifying core regulatory elements within the inflammatory gene network. Prior to model training, sample grouping variables were appropriately encoded to satisfy input format requirements, and gene expression data were standardized to reduce bias due to scale disparities across features. A random forest classifier was then constructed, and feature importance scores were computed to quantify the relative contribution of each gene to the prediction of individual inflammatory markers. Finally, the results were visualized to intuitively highlight the most influential genes, enabling identification of potential upstream regulators involved in RA-associated inflammation and oxidative stress.

The random forest model was selected primarily for its robustness and suitability for small-sample transcriptomic datasets. In contrast to deep learning methods such as neural networks, which typically require large training sets to achieve stable generalization, random forest can maintain high predictive accuracy with limited sample sizes while effectively minimizing the risk of overfitting. Moreover, it possesses strong nonlinear mapping capabilities, allowing the model to capture intricate, nonlinear relationships between gene expression patterns and inflammatory marker levels. In addition to its predictive strengths, random forest models offer inherent interpretability by computing feature importance scores, which is particularly valuable in biological research for identifying potential regulatory genes. Another key advantage is the model’s resilience to missing values and biological noise, making it well-suited for analyzing high-dimensional, sparse, and unstructured omics data.

Model outputs revealed that gene contributions to TNF-α, IL-6, IL-1β, and ROS regulation were heterogeneous. Notably, FADS1 exhibited a markedly higher importance score than other genes in predicting IL-1β levels, suggesting a potentially specific role in IL-1β-mediated inflammatory processes. This may relate to dysregulated lipid metabolism observed in RA pathogenesis, in which FADS1 plays a central role. Other genes, including LBH, CCL19, and P2RY10, also contributed to IL-1β regulation but with comparatively lower importance scores, suggesting auxiliary or synergistic effects (Figure 8c).

Similarly, for TNF-α-associated genes, LBH exhibited the highest importance score, suggesting a central role in TNF-α-mediated inflammatory signaling. TAGAP and CCL19 also demonstrated substantial contributions. TAGAP is involved in T cell activation, while CCL19 regulates inflammatory cell recruitment; both may promote TNF-α expression by modulating immune cell functionality (Figure 8d). In the case of ROS-related genes, FCGR2B showed the highest contribution, followed by TAGAP and CCL19. This pattern implies that ROS production in RA may be influenced by immunoglobulin receptor-mediated signaling and T cell activation pathways. Additionally, TNFRSF14 also displayed high contribution, indicating that T cells may play a pivotal role in regulating oxidative stress responses in the RA microenvironment (Figure 8e). In contrast, for IL-6 regulation, LBH and TAGAP remained top contributors, accompanied by FCGR2B, CCL19, and CD40. These genes are closely associated with B cell activation and pro-inflammatory cytokine release, suggesting their involvement in driving IL-6–dependent inflammatory cascades (Figure 8f).

A comparative analysis of gene contribution patterns across ROS and the three cytokines revealed that FADS1’s importance in IL-1β regulation was disproportionately higher than its contributions to TNF-α, IL-6, or ROS. This suggests a selective role for FADS1 in IL-1β–mediated pathways, likely related to lipid metabolism dysregulation in RA, rather than a universal influence on all inflammatory processes. Interestingly, core regulatory genes such as LBH, TAGAP, CCL19, and FCGR2B demonstrated high contribution scores across multiple inflammatory mediators and ROS, indicating that these genes may function as central nodes within the RA inflammatory regulatory network. In contrast, CD40 and IL2RA showed notable contributions to IL-6 modulation but minimal influence on TNF-α and ROS, implying a B cell–dominated mechanism in IL-6–driven inflammation, whereas T cell–associated pathways may play a more prominent role in regulating TNF-α and oxidative stress. In summary, by applying a random forest model to high-dimensional transcriptomic data, this study identified key regulatory genes underlying inflammatory cytokine production and oxidative stress in RA. LBH, TAGAP, CCL19, and FCGR2B emerged as multi-factorial contributors, potentially serving as critical regulators of disease progression. The unique and dominant role of FADS1 in IL-1β signaling highlights its promise as a target for metabolic-inflammation–linked interventions. Collectively, these findings enrich our understanding of RA pathophysiology and offer valuable targets for refining personalized therapeutic strategies. While transcriptomic profiles, serum cytokines/ROS, and joint-level phenotypes are concordant, definitive protein-level mechanistic regulation (e.g., phospho-signaling dynamics, loss-of-function assays) was not addressed here and represents an important direction for future work. Accordingly, we present pathway-level conclusions as associative rather than causal.

## 4. Conclusions

In this work, systematic evaluation of hollow microneedles-mediated drug delivery was performed to demonstrate the therapeutic potential of localized delivery system by effective delivery of Tofacitinib and NAC separately in a rat model of RA. Histological staining, micro-CT, and inflammatory scoring confirmed that both drugs alleviated synovial inflammation, cartilage damage, and bone loss, with NAC showing stronger effects on ROS suppression. ELISA and transcriptomic analyses revealed that the treatments downregulated pro-inflammatory cytokines and modulated key immune and bone-related pathways. Gene-pathway mapping and correlation analyses identified core inflammatory regulators such as Clec4e, Ccr5, Tlr7, and Nod2, while Ackr4 emerged as a potential protective gene. Random forest modeling further highlighted LBH, TAGAP, CCL19, FCGR2B, and FADS1 as key contributors to inflammatory responses and oxidative stress, offering promising targets for precision therapy. Overall, microneedle-based local drug delivery provides an effective strategy for modulating inflammation and immune imbalance in RA, and integrative transcriptomic and machine learning analyses offer valuable tools for identifying novel therapeutic targets, which could potentially foster treatment progress and enable clinical implementation.

## Data Availability

Data are contained within the article.
